# Ketamine: 50 Years of Modulating the Mind

**DOI:** 10.3389/fnhum.2016.00612

**Published:** 2016-11-29

**Authors:** Linda Li, Phillip E. Vlisides

**Affiliations:** ^1^Department of Internal Medicine, St. Joseph Mercy HospitalAnn Arbor, MI, USA; ^2^Department of Anesthesiology, University of Michigan Medical SchoolAnn Arbor, MI, USA

**Keywords:** ketamine, neuropharmacology, consciousness, anesthesia, functional connectivity, depression, post-traumatic stress disorder

## Abstract

Ketamine was introduced into clinical practice in the 1960s and continues to be both clinically useful and scientifically fascinating. With considerably diverse molecular targets and neurophysiological properties, ketamine’s effects on the central nervous system remain incompletely understood. Investigators have leveraged the unique characteristics of ketamine to explore the invariant, fundamental mechanisms of anesthetic action. Emerging evidence indicates that ketamine-mediated anesthesia may occur via disruption of corticocortical information transfer in a frontal-to-parietal (“top down”) distribution. This proposed mechanism of general anesthesia has since been demonstrated with anesthetics in other pharmacological classes as well. Ketamine remains invaluable to the fields of anesthesiology and critical care medicine, in large part due to its ability to maintain cardiorespiratory stability while providing effective sedation and analgesia. Furthermore, there may be an emerging role for ketamine in treatment of refractory depression and Post-Traumatic Stress Disorder. In this article, we review the history of ketamine, its pharmacology, putative mechanisms of action and current clinical applications.

## Introduction

Fifty years ago, Corssen and Domino (1966) published the first clinical study of ketamine as a human anesthetic. Ketamine produces an unusual state, sometimes referred to as “dissociative anesthesia”, which was a term coined by Domino’s (2010) wife. During this dissociative state, patients might appear awake with preserved airway reflexes and respiratory drive, but they are unable to respond to sensory input (Domino et al., 1965). Ketamine additionally provides excellent analgesia with an impressive safety profile, making it a popular anesthetic induction agent in a variety of patient populations and settings.

Although initially developed as an anesthetic, over the past several decades ketamine has been revealed to have greater potential in the field of medicine. A growing body of literature has demonstrated the clinical value of ketamine across diverse settings, with emerging roles in pain medicine and treatment-resistant depression. Concurrently, efforts to uncover the mechanisms underlying ketamine’s actions are providing researchers with new insights into the relationship between consciousness and anesthesia.

Since the first clinical report in 1966, ketamine has become arguably the most unique anesthetic agent used today and also one of the most promising and exciting in terms of its potential. The purpose of this article is to provide an overview of ketamine’s history, pharmacology, putative mechanisms and clinical applications.

## History

Ketamine’s history begins with phencyclidine, which was first synthesized in 1956 by chemists at Parke Davis Company (Maddox et al., 1965) who discovered ketamine’s unique and fascinating pharmacology. Phencyclidine was capable of causing the appearance of drunkenness in rodents, delirium in dogs, cataleptoid states in pigeons and anesthesia in monkeys (Domino and Luby, 2012). Although demonstrated to be a safe and reliable anesthetic in humans, it also caused an intense, prolonged emergence delirium that ultimately made it undesirable for human use (Greifenstein et al., 1958; Johnstone et al., 1959; Domino and Luby, 2012).

**Figure 1:**
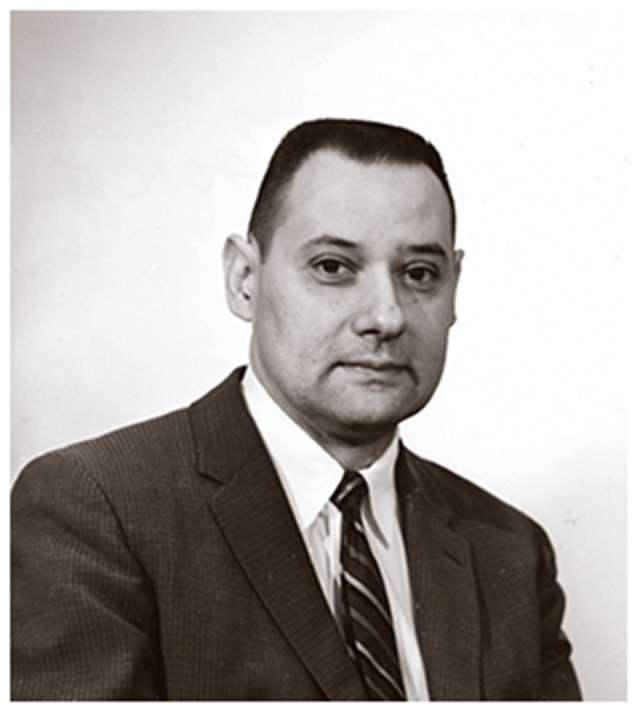
**Dr. Edward Domino as a young faculty at the University of Michigan.** Dr. Domino is now in his 90s, an Emeritus Professor, and still active as a scientist in the field of neuropharmacology. Photograph provided courtesy of the University of Michigan Bentley Historical Library.

Efforts were subsequently redirected towards synthesizing shorter-acting analogs of phencyclidine that would have similar anesthetic potential but cause less emergence delirium. Ketamine, then identified as CI-581, was one such agent developed by Parke Davis consultant and organic chemist Calvin Stevens in 1962 (Domino, 1980). A structural analog at one-tenth the potency of its parent drug phencyclidine, ketamine was subsequently selected for human trials, and the first human anesthetic dose was administered on August 3, 1964 by two University of Michigan professors: Dr. Edward Domino of Pharmacology (Figure 1) and Dr. Guenter Corssen of Anesthesiology. In their initial pharmacological study of ketamine in 20 humans, Domino and Corssen found evidence that the drug could be safe and effective for clinical anesthetic use (Domino et al., 1965). In 1966, they published findings from the first clinical experiences with ketamine, reporting its anesthetic effects in 130 patients, aged 6 weeks to 86 years, undergoing a total of 133 surgical procedures (Corssen and Domino, 1966). They found that ketamine could rapidly produce profound analgesia with a unique state of altered consciousness and a limited duration of effect that could be safely prolonged with repeated administration. They also reported minimal side effects and a lack of severe emergence delirium compared to phencyclidine (Corssen and Domino, 1966). Ketalar (1970) became the first preparation of ketamine approved by the food and drug administration (FDA) for human use.

## Pharmacology

### Structure

**Figure 2:**
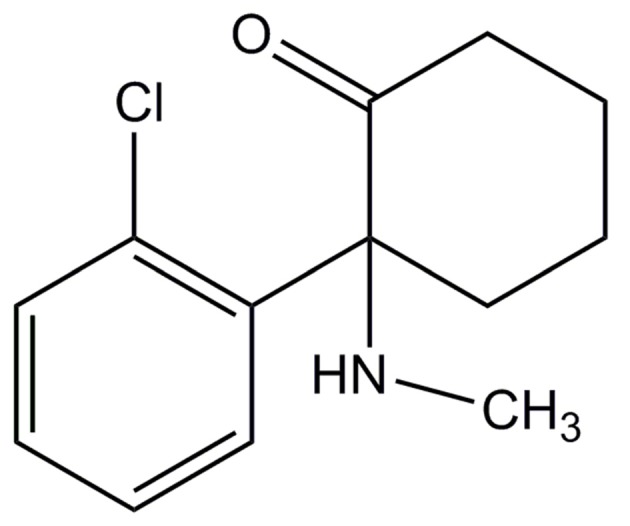
**Structure of Ketamine**.

Ketamine is an arylcycloalkylamine that exists as S(+) and R(−) isomers and is commonly marketed as a racemic mixture of the two (Figure 2). Isolated S(+) ketamine, which is not currently available in the United States but marketed in other parts of the world, has a higher affinity to the binding site on N-methyl-D-aspartate (NMDA)-receptors and produces 3–4 times greater anesthetic potency than the R(−) isomer (White et al., 1985). When used intraoperatively, the S(+) isomer is associated with less cardiac stimulation, less spontaneous motor activity, better analgesia, more rapid recovery, fewer psychotomimetic side effects, and a decreased incidence of emergence delirium (White et al., 1980, 1985). In the more recent use of ketamine as an antidepressant, mouse studies have shown the R(−) isomer to be more potent and with less side effects than the S(+) isomer (Zhang et al., 2014).

### Pharmacokinetics and Pharmacodynamics

#### Administration and Bioavailability

Soluble in both water and lipids, ketamine can be safely administered through multiple routes: intravenous (IV), intramuscular (IM), oral, nasal, rectal, subcutaneous and epidural. IV administration is 100% bioavailable and considered the ideal route of administration. However, in certain settings such as emergencies or with uncooperative patients, IM ketamine is commonly used and has only a slightly lower bioavailability of 93% (Clements et al., 1982). A summary of ketamine’s common routes of administration and their respective pharmacologic profiles is provided in Table 1.

**Table 1:** **Basic pharmacologic profiles of ketamine**.

Route	Dose*	Bioavailability	*T_max_*(minutes)	References
IV	1–4.5 mg/kg	100%	3	Weber et al. (2004)
IM	6.5–13 mg/kg	93%	5–10	Clements et al. (1982)
Intranasal	0.5–1 mg/kg	8–45%^†^	10–20	Yanagihara et al. (2003)
				Huge et al. (2010)
				Andolfatto et al. (2013)
				Yeaman et al. (2014)
PO	0.25–0.5 mg/kg	17–29%^†^	30	Grant et al. (1981)
				Clements et al. (1982)
				Chong et al. (2009)
				Blonk et al. (2010)
				Rolan et al. (2014)
Rectal	9–10 mg/kg	11–25%	30–45	Idvall et al. (1983)
				Pedraz et al. (1989)
				Malinovsky et al. (1996)

*Dosages may vary depending on clinical setting; anesthetic doses provided per manufacturer labeling for intravenous (IV) and intramuscular (IM) routes. Representative analgesic doses and bioavailability data are outlined for intranasal, oral (PO) and rectal routes. ^†^Intranasal and PO absorption vary significantly. *T_max_*, time to maximum plasma concentration; mg, milligram; kg, kilogram.

Other routes of administration are less commonly used but exist as feasible options nonetheless. Intraosseous ketamine, for instance, has slightly slower anesthetic onset compared to IV administration (71.3 s and 56.3 s, respectively; Aliman et al., 2011) but may be used in emergency settings. Ketamine can also be administered intranasally, though it has a lower bioavailability of approximately 45% (Yanagihara et al., 2003) and can vary depending on the amount absorbed through nasal mucosa and the amount swallowed. Due to ketamine’s extensive first-pass metabolism, rectal and oral formulations have limited bioavailability with relatively high concentrations of the active (but less-potent) metabolite norketamine (Malinovsky et al., 1996; Chong et al., 2009; Rolan et al., 2014). Historically, these routes are used infrequently in humans, though increasing efforts are being made to develop suitable oral and sublingual formulations given the recent move towards using low-dose ketamine for pain and depression in the outpatient setting (Chong et al., 2009; Rolan et al., 2014).

#### Distribution, Metabolism and Excretion

Due to ketamine’s high lipid solubility and relatively limited protein binding, it is rapidly taken up by the brain and redistributed, with a distribution half-life of only 10–15 min (Wieber et al., 1975; Domino et al., 1984). Ketamine has a large volume of distribution of nearly 3 L/kg (Clements and Nimmo, 1981). Once in the body, ketamine undergoes liver metabolism to several metabolites (Clements and Nimmo, 1981). Of note, metabolism through cytochrome systems forms the active metabolite norketamine, which retains anesthetic activity but at one-third the potency of ketamine (Cohen and Trevor, 1974; Domino et al., 1984). Inactive ketamine conjugates and metabolites are renally excreted (Wieber et al., 1975), and elimination half-life is 2–3 h (Domino et al., 1984).

### Dosing

Ketamine’s wide therapeutic range makes it one of the safest anesthetics available. General anesthesia can be induced with both IV and IM routes (Table 1) and maintained with repeated doses of 0.5–1 mg/kg (Domino et al., 1984). Good analgesia and sedation can also be achieved at subanesthetic doses (e.g., 0.2–0.8 mg/kg IV, 2–4 mg/kg IM), and infusions at subanesthetic doses (e.g., 0.5 mg/kg/h) may also provide continuous sedation and analgesia (Allen and Macias, 2005; Miller et al., 2011).

### Systemic Effects

#### Cardiovascular

At both subanesthetic and anesthetic doses, ketamine is predominantly a sympathomimetic, producing increased arterial pressures and heart rate (Corssen and Domino, 1966) through direct stimulation of central nervous system structures (Traber et al., 1970). At higher doses (e.g., 20 mg/kg), however, ketamine also acts as direct myocardial depressant (Traber et al., 1968), and in the setting of compromised autonomic control (e.g., spinal cord transection, catecholamine depletion), these depressive effects may be unmasked. Ketamine also causes direct relaxation of vascular smooth muscle, though due to its sympathetically-mediated vasoconstriction, it has a relatively stable net effect on systemic vascular resistance (Diaz et al., 1976; Akata et al., 2001; Jung and Jung, 2012).

#### Pulmonary

Ketamine does not cause clinically significant respiratory depression in patients (Corssen and Domino, 1966), though arterial hypoxemia following rapid IV infusion of ketamine has been reported (Zsigmond et al., 1976). In fact, low-dose ketamine may actually stimulate respiration and is observed to produce higher flow-rates, respiratory rates and duty-cycle (inspiratory time divided by total respiratory cycle time) in animal models (Eikermann et al., 2012). Ketamine is also unique in its ability to preserve upper airway reflexes during anesthesia, uncoupling the loss of consciousness with the loss of upper airway dilator muscle activity (Eikermann et al., 2012). Ketamine further increases genioglossus muscle activity (Eikermann et al., 2012); this elevates and pushes the tongue forward, increasing upper airway diameter to further prevent its collapse (Oliven et al., 2003).

Despite the fact that a patent airway is usually maintained during exposure to ketamine, attention to airway protection remains essential, as partial obstruction and aspiration are still possible. Ketamine may increase salivary secretions (Corssen and Domino, 1966) and potentially increase the risk of laryngospasm, but this is rarely reported (Green et al., 2010). Other respiratory effects of ketamine include bronchodilation, likely through vagolytic and other neurally mediated mechanisms (Brown and Wagner, 1999). At high doses, ketamine may also directly affect airway smooth muscle, but this effect is unlikely to be of clinical importance (Brown and Wagner, 1999).

#### Neurological

Because ketamine increases cerebral metabolism, it can potentially increase intracranial pressure (ICP) and has been used cautiously in patients with space-occupying cerebral lesions brain injury (Gardner et al., 1971; Shaprio et al., 1972; Wyte et al., 1972). However, when used in combination with propofol or midazolam, effects on cerebral perfusion are comparable to other commonly used, opioid-based combinations (Bourgoin et al., 2003, 2005; Wang et al., 2014). Ketamine has also been safely used in patients with elevated ICP while helping to maintain optimal hemodynamic profiles (Bourgoin et al., 2003; Schmittner et al., 2007). In fact, in some cases, ketamine’s cerebral effects may be neuroprotective and potentially beneficial for brain trauma patients (Albanèse et al., 1997; Bar-Joseph et al., 2009). This includes ketamine’s inhibition of spreading cortical depolarizations after traumatic brain injury (TBI), an effect that may attenuate the extension of ischemic damage to healthier peri-ischemic tissue (Hertle et al., 2012).

### Side Effects, Toxicities, Interactions and Abuse

Ketamine has multiple dose-dependent side effects, though most of which are self-resolving. Adverse effects include hypersalivation, hyperreflexia and transient clonus (Corssen and Domino, 1966). Ketamine may also cause vestibular-type symptoms including dizziness, nausea and vomiting. Cardiopulmonary toxicity is rare, with effects limited to those caused by the transient sympathetic activation such as tachycardia, hypertension and palpitations (Weiner et al., 2000; Strayer and Nelson, 2008). Given ketamine’s wide therapeutic range, death by overdose is rare and usually involves other intoxicants or in the setting of trauma (Moore et al., 1997; Gill and Stajic, 2000).

The psychoactive properties associated with ketamine limit widespread clinical use. Even at subanesthetic doses (i.e., 0.1–0.4 mg/kg; Krystal et al., 1994), patients may experience perturbing dissociative symptoms. One study described ketamine at such doses producing four main psychological effects (Pomarol-Clotet et al., 2006): (1) a feeling of intoxication, comparable to the effects of other anesthetics and sedatives; (2) perceptual alterations in visual, auditory and somatosensory domains concomitant with symptoms of depersonalization or derealization; (3) referential ideas and delusions, often of misinterpretation and thought disorder; and (4) negative symptoms such as poverty of speech.

More recently, cystitis and various lower urinary tract pathologies (e.g., detrusor over-activity) have also been reported in long-term ketamine users (Chu et al., 2008; Tsai et al., 2009). Ketamine abuse is also associated with gastrointestinal symptoms, including biliary dysfunction, epigastric pain and hepatic injury (Poon et al., 2010; Lo et al., 2011; Wong et al., 2014; Yu et al., 2014), though these adverse effects may be reversible with abstinence (Zhou J. et al., 2013; Wong et al., 2014; Yu et al., 2014).

While ketamine’s psychedelic effects limit clinical use, they have made ketamine a popular recreational drug. At lower doses, stimulant effects predominate, and users experience mild dissociation with hallucinations and a distortion of time and space. Higher doses induce more severe, schizophrenia-like symptoms and perceptions that are completely separate from reality (Wolff and Winstock, 2006; Niesters et al., 2014). Although these effects resolve approximately 2 h after acute ketamine use, long-term use can cause more pronounced and persistent neuropsychiatric symptoms, including schizophrenia-like symptoms, cognitive impairment and poor psychological well-being (Morgan et al., 2009, 2010; Liu et al., 2016).

Finally, given its CNS modulatory activity, ketamine should be used cautiously with other drugs that alter mood and perception, including alcohol, opioids, benzodiazepines and cannabis. Ketamine metabolism involves cytochrome P450 enzymes (Hijazi and Boulieu, 2002), and thus, concomitant use with drugs that inhibit cytochrome P450 metabolism may lead to inhibited ketamine metabolism and supratherapeutic toxicity.

## Proposed Mechanisms of Actions

### Molecular Targets

Unlike the IV and inhaled anesthetics in common clinical use, ketamine is not thought to act primarily through the potentiation of gamma-aminobutyric acid (GABA) transmission. Early work on the mechanisms of ketamine found that it reduced neuronal excitation by NMDA, the agonist for which the glutamatergic receptor type is named (Anis et al., 1983). Ketamine was found to block excitatory postsynaptic potentials in rat cortical pyramidal cells (Thomson et al., 1985) and frog spinal cord neurons (Martin and Lodge, 1985) in a manner consistent with NMDA antagonism; these data were supported by studies in the central nervous system of the lamprey (Yamamura et al., 1990). Subsequently, mouse models with altered NMDA receptor subunits showed attenuated responses to ketamine (Petrenko et al., 2004; Sato et al., 2004). In fact, ketamine-mediated NMDA receptor antagonism of GABAergic inhibitory interneurons is a postulated mechanism of disinhibition and psychosis (Homayoun and Moghaddam, 2007; Brown et al., 2011).

Ketamine non-competitively antagonizes the NMDA receptor by at least two distinct mechanisms: as an open-channel blocker, binding to a site within the channel pore to occlude the channel and reduce mean open time, and through an allosteric mechanism to decrease channel opening frequency (Orser et al., 1997). Ketamine also has a slow off-rate (86% trapping) and is an example of a high-trapping antagonist of the NMDA receptor, similar to MK-801 (dizocilpine, nearly 100% trapping). Thus, even after glutamate dissociates from the NMDA receptor, ketamine remains trapped in the closed ion channel and causes continued blockade. Conversely, NMDA receptor antagonists with a fast off-rate (low-trapping), such as memantine, (50–70% trapped) escape the channel before it closes, producing less blockade of physiological NMDA function. This results in fewer side effects (e.g., sedative or psychotomimetic) and an NMDA antagonist without “appreciable anesthetic effects” (Sleigh et al., 2014).

In addition to NMDA-antagonism, ketamine acts on a wide-range of other targets, contributing to its unique effects and uses (Table 2). For instance, ketamine’s relaxant effect on airway smooth muscle has been attributed to its inhibition of L-type voltage-dependent Ca^2+^ channels (Yamakage et al., 1995). Inhibition of calcium channels may also contribute to observed psychodysleptic effects such as dysphoria, psychosis, altered perception and impaired verbal fluency (Baum and Tecson, 1991). A block on monoamine transport systems is also thought to contribute to psychotomimetic and sympathomimetic effects (Nishimura et al., 1998), and recently, a compelling case has been made that hyperpolarization-activated cyclic nucleotide (HCN) channels—sometimes referred to as neuronal pacemaker channels—significantly contribute to ketamine-induced hypnosis (Chen et al., 2009; Zhou C. et al., 2013). These channels may mediate the hypnotic effects of volatile anesthetics as well (Zhou et al., 2015). Although ketamine appears to play a role in opioid potentiation (Finck and Ngai, 1982; Smith et al., 1987; Pacheco et al., 2014), antinociceptive effects mediated by opioid receptors may vary based on receptor subtype (Mikkelsen et al., 1999; Pacheco et al., 2014). Inhibition of serotonin reuptake is another suggested as a mechanism by which ketamine may confer analgesic effects (Martin et al., 1982), and ketamine’s block of large-conductance *K*_Ca_ channels (BK channels) preferentially suppresses spinal microglia hyperactivation after nerve injury and may explain its potent effects on neuropathic pain (Hayashi et al., 2011). More recently, a novel mechanism for activation of α-amino-3-hydroxy-5-methyl-4-isoxazole propionic acid (AMPA) receptors by the (R,S)-ketamine metabolite (2S,6S;2R,6R)-hydroxynorketamine has been implicated in the rapid, antidepressant-like properties observed with ketamine (Zanos et al., 2016). Gonadal hormones, such as estrogen and progesterone, may also potentiate the rapidity and potency of ketamine’s antidepressant effects, as demonstrated in preclinical models (Carrier and Kabbaj, 2013; Franceschelli et al., 2015; Hashimoto, 2016). Lastly, ketamine interaction with GABA receptors has been implicated in various clinical pathologies, including obsessive-compulsive disorder (Rodriguez et al., 2015), depression (Perrine et al., 2014) and learning disabilities following chronic exposure (Tan et al., 2011).

**Table 2:** **Receptor and channel targets of ketamine and related clinical effects**.

**Antagonism/Inhibition**
NMDA receptors	• Dissociative anesthesia, amnesia (Oye et al., 1992) • Inhibited sensory perception (Oye et al., 1992) • Analgesia (Oye et al., 1992)

HCN channels	• Hypnosis (Chen et al., 2009; Zhou C. et al., 2013)
Calcium channels (L-type voltage-dependent)	• Negative cardiac inotropy (Baum and Tecson, 1991) • Airway smooth muscle relaxation (Yamakage et al., 1995)
Voltage-gated sodium channels	• Decreased parasympathetic activity (Irnaten et al., 2002) • Local anesthetic effect (Frenkel and Urban, 1992; Haeseler et al., 2003)
BK channels	• Analgesic effects on neuropathic pain (Hayashi et al., 2011)

**Agonism/Activation**

Opioid receptors (particularly μ, δ)	• Central antinociception (Finck and Ngai, 1982; Pacheco et al., 2014)
AMPA receptors	• Rapid antidepressant effects (Zanos et al., 2016)
GABA_A_ receptors	• Anesthetic properties (Irifune et al., 2000)

*NMDA, N-methyl-D-aspartate; HCN, Hyperpolarization-activated cyclic nucleotide; BK, Large-conductance potassium channels; AMPA, α-amino-3-hydroxy-5-methyl-4-isoxazolepropionic acid; GABA_A_R, γ-aminobutyric acid A receptor*.

Not surprisingly, ketamine’s immediate effects on its various targets cause altered downstream processes. Ketamine’s block of Ca^2+^ influx through NMDA antagonism may, for instance, lower the activity of protein kinase C (PKC) and other intracellular signals, which could then induce altered protein phosphorylation or signaling. In rodent models, systemic ketamine administration suppresses immediate early gene expression (zif/268, c-fos, junB, fosB, c-jun, junD) in the cortex and hippocampal dentate gyrus after mechanical brain injury (Belluardo et al., 1995). It also decreases NMDA receptor 1 phosphorylation (Mei et al., 2010) and affects mRNA expression implicated in rodent models of hyperalgesia (Ohnesorge et al., 2013). Ketamine acutely increases hippocampal proteins brain-derived neurotrophic factor (BDNF; Garcia et al., 2008; Yang et al., 2013) and mammalian target of rapamycin (mTOR; Yang et al., 2013), which may also help to explain the mechanism for its rapid antidepressant effects. Indeed, investigation into ketamine’s antidepressant effects have led to several reports that ketamine may affect various brain regions through epigenetic mechanisms, such as histone deacetylase modulation (Reus et al., 2013; Choi et al., 2015) and increased BDNF mRNA expression (Duman and Voleti, 2012).

### Systems Neuroscience

At the systems neuroscience level, ketamine’s mechanisms are markedly distinct from drugs such as propofol or the halogenated ethers. Unlike most anesthetics, ketamine does not appear to activate the sleep-promoting ventrolateral preoptic nucleus of the hypothalamus; rather, ketamine activates subcortical wake-promoting nuclei (Lu et al., 2008). This is paralleled in ketamine’s unique neurochemistry. Unlike most other inhaled and IV anesthetics, ketamine increases levels of the arousal-promoting neurotransmitter acetylcholine in the cortex (Pal et al., 2015) and it depends, in part, on noradrenergic transmission for its full effect (Kushikata et al., 2011). Ketamine is the only sedative-hypnotic that maintains or increases thalamic metabolism (Langsjo et al., 2005). Neurophysiologically, ketamine—unlike propofol and sevoflurane—increases electroencephalographic activity around 40 Hz (Lee et al., 2013), but—like other anesthetics—suppresses high gamma activity (Pal et al., 2015) and increases delta power (Lee et al., 2013).

Ketamine is also known to alter neuromodulation of various neurotransmitter systems. It reduces cholinergic neurotransmission by acting as a non-competitive and voltage-dependent inhibitor of nicotinic acetylcholine ion channel receptors, particularly in β1 subunit-containing receptors (Yamakura et al., 2000). It appears to reduce acetylcholine release in the medial pontine reticular formation (mPRF), which may in part explain its ability to alter arousal and breathing (Lydic and Baghdoyan, 2002). Unique to most other anesthetics, ketamine produces stimulatory effects on noradrenergic neurons in the medial prefrontal cortex (mPFC; Kubota et al., 1999), and more recently, it has also been shown to raise ACh levels in the PFC during anesthetic dosing (Pal et al., 2015). Despite increased cholinergic tone in the PFC, however, high-frequency gamma activity and cortical coherence were both suppressed, suggesting that ketamine-induced unconsciousness is characterized by dissociation of cholinergic tone and cortical activation, possibly mediated by suppression of the NMDA receptors on both pyramidal and inhibitory neurons. Thus, with preserved activation of subcortical arousal nuclei and increased cholinergic tone, ketamine-induced loss of consciousness may occur via higher-level mechanisms, as evidenced by fragmented cortical coherence and attention of high-frequency gamma activity (Pal et al., 2015). Disrupted cortical communication and information transfer may in fact be a common mechanism of loss of consciousness across diverse pharmacological classes of anesthetics (Lee et al., 2013), as will be discussed further below.

### Network Connectivity

**Figure 3:**
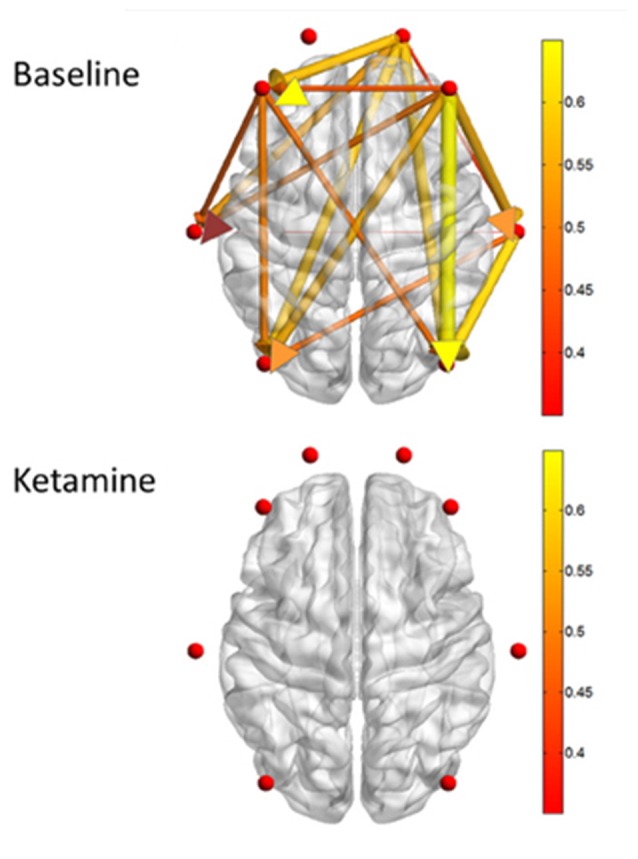
**Measures of directed connectivity after induction with Ketamine, adapted from Blain-Moraes et al. (2014).** Graphical depiction of dominant feedback connectivity in the waking state that is neutralized after ketamine induction. Please see original article (Blain-Moraes et al., 2014) for additional information.

Despite the differences of ketamine’s actions at the molecular and systems neuroscience levels, there appears to be a common network-level effect that might explain the common functional outcome of lost responsiveness induced by ketamine and the primarily GABAergic anesthetics. Ketamine has been found to functionally disrupt corticocortical connectivity with anesthetic dosing, and this pattern seems to follow a frontal-to-posterior direction (Figure 3; Lee et al., 2013; Blain-Moraes et al., 2014; Schroeder et al., 2016). This frontal-to-posterior disrupted cortical connectivity has been proposed as a mechanism of general anesthesia, having been demonstrated across a broad range of anesthetic drug classes during loss of consciousness (Lee et al., 2013). Previous electroencephalographic studies have used indirect, surrogate measures of information transfer—such as transfer entropy and phase lag index—to demonstrate disrupted connectivity during anesthetic-induced loss of consciousness (Lee et al., 2013; Blain-Moraes et al., 2014). Bonhomme et al. (2016) presented functional MRI data from human volunteers whereby ketamine disrupted cortical connectivity between frontal and posterior cortices, while auditory and visual network integrity was maintained. A recent study used intracranial recordings to demonstrate direct, structural interruption of corticocortical sensory information transfer during ketamine-induced anesthesia in macaque monkeys (Schroeder et al., 2016). During anesthesia, somatosensory information transfer was maintained through thalamocortical pathways, though corticocortical information transfer was inhibited. This study showed direct evidence of inhibited information transfer along structurally connected cortical pathways. Thus, collectively, there is accumulating evidence that ketamine disrupts corticocortical information transfer in the frontal-to-posterior direction while preserving sensory network function during anesthesia. These findings contribute to a higher-order, network-level understanding of the neural correlates of consciousness and also shed further insight into the “dissociative” anesthetic nature of ketamine.

Subanesthetic ketamine administration has recently been shown to alter functional connectivity patterns as well, with possible mechanistic implications for depression. Wong et al. (2016) found that subanesthetic ketamine administration disrupted functional connectivity between the subgenual anterior cingulate cortex, which is involved with the modulation of mood (Davey et al., 2012), and a network cluster involving the thalamus, hippocampus and the retrosplenial cortex. Using a ketamine infusion dose often used for the treatment of depression, Muthukumaraswamy et al. (2015) demonstrated reduced functional connectivity in frontoparietal networks concomitant with an increase in blissful feelings. Thus, modulation of brain connectivity patterns might also provide a network-level mechanism for ketamine’s effects on depression. As patients in these studies were not reported to lose consciousness, there may be a dose-dependent component to the disruption of functional connectivity.

## Clinical Uses in Medicine

Ketamine has enjoyed expanded clinical relevance since its early development and is now being actively used or explored in a number of clinical fields (Table 3).

**Table 3:** **Summary of clinical uses for ketamine**.

Anesthesia	Analgesia and sedation		Psychiatry and neuroscience
*Advantageous settings*: • Hemodynamic instability • Pediatric patients • Uncooperative patients • Traumatic brain injury • Bronchospasm • Battlefield/Mass casualty	*Acute settings*: • Procedures • Burns • ED Agitation/Pain • Post-operative pain	*Chronic settings*: • Cancer pain • CRPS • Phantom limb pain • Fibromyalgia • Ischemic pain • Migraines	*Emerging use*: • Depression • Suicidal ideation • PTSD *Modeling*: • Schizophrenia • Consciousness

*ED, emergency department; CPRS, complex regional pain syndrome; PTSD, post-traumatic stress disorder. Please see text (“Clinical Uses in Medicine” Section) for associated references*.

### Anesthesia

Ketamine’s rapid onset, safety, and hemodynamic stability have made it a useful anesthetic induction agent, especially in certain patient populations or settings where its characteristics are particularly advantageous.

#### Hemodynamic Instability

Ketamine’s effects of sympathetic activation may make it beneficial for use in hemodynamically unstable patients (e.g., traumatic injury, septic shock). In septic patients requiring emergency intubation, ketamine may also be a safer alternative to etomidate, which may cause adrenal insufficiency and is associated with higher in-hospital morbidity in such patient populations (Jabre et al., 2009). However, caution should still be observed, as the cardiac depressant effects may hasten cardiovascular compromise in catecholamine-depleted states (Waxman et al., 1980; Dewhirst et al., 2013).

#### Pediatrics

The ability to administer ketamine intramuscularly has made it advantageous for patients in which IV administration may be difficult, including neonates, infants and young children. Together with the ease of administration, efficacy and safety profile in children, ketamine has become one of the most commonly used drugs for procedural sedation and analgesia for children in emergency departments (ED; Haley-Andrews, 2006; Bhargava and Young, 2007). However, anecdotal observations suggest a higher risk of airway complications with ketamine in infants less than 3 months of age (Green and Johnson, 1990; Green et al., 2011), likely due to infant-specific differences in airway reactivity and anatomy rather than ketamine itself. Furthermore, although emergence reactions in children and teenagers are rare and typically mild, risk factors for recovery agitation have emerged. Two clinically pertinent risk factors are: (1) low IM ketamine dosing (<3.0 mg/kg), which may not provide suitable analgesia but still increases agitation; and (2) unusually high IV ketamine dosing (>2.5 mg/kg), which increases the risk of airway complications and emesis (Green et al., 2009). Finally, the use of ketamine as an opioid-sparing agent in children has recently been challenged by a large meta-analysis (Michelet et al., 2016), though this may have been from lack of power given the studies available. Further investigation into ketamine’s effects in children is certainly warranted.

#### Traumatic Brain Injury (TBI)

Despite historic concerns that ketamine may cause harmful increases in ICP, recent reports have challenged this with evidence that ketamine can be safely and effectively used in patients with head injuries or risk of intracranial hypertension (Bourgoin et al., 2003, 2005; Wang et al., 2014). In fact, ketamine may have beneficial effects, including protection against seizures, cerebral ischemia or secondary brain injury related to hypotension (Albanèse et al., 1997; Bar-Joseph et al., 2009).

#### Bronchospasm

Ketamine’s bronchodilating properties make it an attractive anesthetic induction agent for patients with active bronchospasm. Of note, however, ketamine may predispose patients to laryngospasm through its stimulation of copious secretions, but the incidence of this appears to be relatively rare (Green et al., 2010).

#### Trauma Medicine

Upon being marketed as a human anesthetic in the 1970s, ketamine quickly became a popular battlefield anesthetic and continues to be used in military conflict (Mercer, 2009). Its pharmacological properties and ease of administration allow it to be a safe and effective option for anesthetic use in pre-hospital settings, including mass casualty events (Ashkenazi et al., 2005). Moreover, ketamine’s emerging role in affective disorders may have the potential to protect patients in these settings from developing stress-induced disorders (Brachman et al., 2016).

### Analgesia and Sedation

Unlike most other agents, ketamine offers the important advantage of being able to provide both profound analgesia and adequate sedation without significantly compromising airway reflexes or respiratory function (Corssen and Domino, 1966). It is thus often used in acute clinical settings, though there is a growing interest in its role as a chronic therapeutic agent as well.

#### Acute Setting

##### Procedural sedation

Ketamine is frequently used in the ED for procedural sedation. IM administration has made it an especially popular choice for sedation in children who may otherwise be uncooperative. Although not as frequently used in adults for this purpose due to an increased likelihood of emergence delirium (1–2% in children vs. 10–20% in adults; Strayer and Nelson, 2008), this can effectively be reduced with benzodiazepine administration (Dundee and Lilburn, 1978; Perumal et al., 2015) or with the co-administration of propofol (Willman and Andolfatto, 2007; Andolfatto and Willman, 2010).

##### Burn medicine

Historically, ketamine has played a prominent role in burn care protocols, providing effective analgesia and sedation for burn patients who must undergo debridements, grafts, and repeated dressing changes (Demling et al., 1978; Hondorp, 1987; Canpolat et al., 2012; Kundra et al., 2013) without compromising the airway or respiratory function. Furthermore, the ability to administer ketamine intramuscularly and even orally provides an additional advantage in burn patients who have extensive scarring that might make IV administration challenging.

##### Acute agitation

Ketamine is also used to treat acutely agitated and violent patients in the ED. Even among agitated patients who are intoxicated, ketamine does not appear to have any major adverse effects on physiological stability (Hopper et al., 2015). However, additional pharmacological treatment is often required in these patients, suggesting that ketamine may be useful only for initial control of severe agitation in this setting (Hopper et al., 2015).

##### Acute pain

More recently, low-dose ketamine infusions have been advocated to provide pain relief in the ED. Administering a 15 mg bolus of IV ketamine followed immediately by a continuous infusion at 20 mg/h for 1 h may significantly improve pain scores while maintaining stable vital signs and high patient satisfaction (Ahern et al., 2015).

##### Postoperative pain

The use of ketamine infusions has been shown to be an opiate-sparing technique in managing post-operative pain following a variety of surgeries, including abdominal (Guillou et al., 2003; Webb et al., 2007; Zakine et al., 2008; Kaur et al., 2015), thoracic (Michelet et al., 2007; Nesher et al., 2008, 2009; Chazan et al., 2010), orthopedic (Adam et al., 2005; Kollender et al., 2008; Cha et al., 2012; Akhavanakbari et al., 2014), spinal (Kim et al., 2013) and gynecological (Sen et al., 2009; Suppa et al., 2012). However, others have not observed ketamine to have significant clinical benefits or opioid-sparing effects in postoperative pain management (Jensen et al., 2008; Sveticic et al., 2008; Reza et al., 2010; Yeom et al., 2012). These discrepant results may be explained by variation in dosing strategies, patient profiles and other co-administered analgesics.

#### Chronic Setting

##### Cancer pain

Ketamine’s potentiation of opioid analgesia and opioid-sparing effect may be useful in cancer patients who otherwise require a high-dose of opioids, although as stated above, there is currently conflicting evidence surrounding ketamine’s effects on opioid requirements. While ketamine’s use as an adjunct analgesic has been demonstrated by randomized control trials (Yang et al., 1996; Mercadante et al., 2000) and smaller studies and case reports (Fine, 1999; Tarumi et al., 2000; Kannan et al., 2002; Amin et al., 2014), the evidence remains inconclusive, as others have not found any net clinical benefit by using ketamine as a part of the analgesic regimen in cancer pain (Hardy et al., 2012; Salas et al., 2012). As above with postoperative pain, inconsistent findings may result from variations in study design.

##### Non-cancer pain

Ketamine is increasingly used as an adjunct in treating chronic pain states and appears to provide analgesia through its direct NMDA-receptor antagonism as well as modulation of descending inhibitory paths of pain often implicated in chronic pain states (Niesters et al., 2013). For example, ketamine has been used as an analgesic in patients with complex regional pain syndrome (CPRS: Correll et al., 2004; Finch et al., 2009; Schwartzman et al., 2009; Sigtermans et al., 2009). IV and oral ketamine have also been reported to alleviate symptoms of phantom limb pain (Shanthanna et al., 2010; Mitra and Kazal, 2015), though one case report demonstrated exacerbation of pain after IV ketamine, possibly due to ketamine-induced hallucinations (Sakai and Sumikawa, 2014). Ketamine can also attenuate key mechanisms in fibromyalgia patients, such as muscle and referred pain (Graven-Nielsen et al., 2000); however, these infusions may only provide short-term analgesic relief (Noppers et al., 2011). Ketamine is also an effective analgesic in a variety of chronic pain cases, such as ischemic pain (Liman et al., 2015), migraine with aura (Afridi et al., 2013), breakthrough pain in chronic pain states (Carr et al., 2004), and in patients with developed opioid tolerance or previous opiate abuse (Chazan et al., 2008; Dahi-Taleghani et al., 2014).

### Psychiatric Uses

#### Depression

There has been surging interest in the use of ketamine as a potential therapeutic agent for affective disorders, particularly depression. Even a single-dose of ketamine may cause rapid antidepressant effects in otherwise treatment-resistant cases of bipolar (DiazGranados et al., 2010b; Ibrahim et al., 2011; Kantrowitz et al., 2015) and major depression (Zarate et al., 2006; Murrough et al., 2013). Remarkably, this also includes the acute reduction of suicidal ideation (DiazGranados et al., 2010a; Larkin and Beautrais, 2011; Zigman and Blier, 2013; Murrough et al., 2015). Recent neuroimaging studies support potential anti-anhedonic and anti-depressant effects, demonstrating its ability to alter glucose metabolism in regions implicated in mood disorders (Lally et al., 2014, 2015; Nugent et al., 2014). Repeated ketamine doses may improve depressive symptoms comparable to—and perhaps even more rapidly than—electroconvulsive therapy (ECT; Ghasemi et al., 2014), and it may even be successful in treating ECT-resistant depression (Ibrahim et al., 2011). Despite its observed promising antidepressant effects, however, more rigorous investigation is needed to establish its clinical use as an antidepressant. The current evidence is limited by bias, small sample sizes, and limited data on important cofounding variables. In fact, a recent Cochrane Review determined that the efficacy of ketamine as an antidepressant may be limited beyond 1 week (McCloud et al., 2015).

#### Post-Traumatic Stress Disorder (PTSD)

One of the newer applications of ketamine is its role as a potential treatment for Post-Traumatic Stress Disorder (PTSD), although studies examining this remain limited (Feder et al., 2014; Donoghue et al., 2015). For instance, Feder et al. (2014) found that ketamine may reduce symptom severity of PTSD more rapidly than midazolam; however, they did not exclude previously depressed patients, and the observed results may have been due—in part—to ketamine’s known antidepressive effects. A case reported by Donoghue et al. (2015) describing ketamine-induced remission of PTSD and disruptive symptoms in a child similarly provides inconclusive evidence for effects of ketamine specific to PTSD. While it is postulated that ketamine may be useful in preventing the development of PTSD through the induction of stress resilience (Brachman et al., 2016), more research is clearly needed to better define ketamine’s effects on PTSD.

#### Models of Schizophrenia

Since its discovery, ketamine has been observed to produce symptoms similar to those of schizophrenia. As a result, researchers have used these drugs extensively as models to study schizophrenia. While it now appears that overlaps in symptoms and even receptor effects are insufficient to explain the complex neuropathology of schizophrenia, ketamine and has undoubtedly facilitated and stimulated research efforts into understanding schizophrenia (Domino and Luby, 2012).

## Conclusions and Future Directions

When the first clinical use of ketamine was reported in 1966 in Anesthesia and Analgesia, it quickly became a popular induction agent among anesthesiologists. Now, 50 years later, ketamine is increasingly being used beyond the operating room, demonstrating clinical utility in the fields of emergency medicine, critical care medicine, pain medicine and psychiatry.

The growing interest of ketamine in a range of settings and patient populations may reflect an impressive benefit-to-risk ratio. Unique compared to other anesthetic agents, ketamine produces potent anesthesia, sedation and analgesia while maintaining cardiopulmonary stability and airway patency. Ketamine offers flexible options for administration, and the adverse psychological effects are often transient and either prevented by or alleviated with premedication or combined use with other agents. Nonetheless, the psychoactive effects often remain the limiting factor for its expanded clinical use, and just as ketamine was originally developed to reduce the emergence delirium that prevented phencyclidine’s use in humans, researchers have now moved towards developing shorter acting analogs of ketamine to further reduce the psychogenic effects (Harvey et al., 2015).

Scientifically, investigators have taken advantage of ketamine’s complex molecular and neurophysiological mechanisms of action to gain further insights into the neural correlates of consciousness. Ketamine appears to inhibit information transfer in cortical networks (Bonhomme et al., 2016; Schroeder et al., 2016), which may be a shared mechanism by which diverse anesthetics induce unconsciousness (Lee et al., 2013). Perturbations in cortical network connectivity also correlate with pathological brain states, such as depression (Muthukumaraswamy et al., 2015; Nugent et al., 2016), and ketamine-induced alterations in connectivity patterns may subserve its antidepressant effects (Muthukumaraswamy et al., 2015; Nugent et al., 2016). Indeed, ketamine is being used as a tool for probing the mind to further inform our neurobiological framework of consciousness and altered brain states, and lines of investigation are actively ongoing (Mashour, 2016).

Over the past 50 years, countless patients have benefited greatly from ketamine. We anticipate many more exciting discoveries in the next 50 years of investigating its clinical applications and mechanisms of modulating the mind.

## Author Contributions

Both authors contributed to the writing of the manuscript, approved the final version to be published, and are accountable for manuscript accuracy and integrity. LL: conception, design and drafting of the initial manuscript. PEV: critical manuscript review and revision of all final content.

## Funding

PEV is supported by the National Institutes of Health, Bethesda, MD, USA, Grant T32GM103730. Funding also provided by the Department of Anesthesiology, University of Michigan Medical School.

## Conflict of Interest Statement

The authors declare that the research was conducted in the absence of any commercial or financial relationships that could be construed as a potential conflict of interest.
